# Effect of Ball Burnishing on Fretting at Elevated Temperatures

**DOI:** 10.3390/ma17235960

**Published:** 2024-12-05

**Authors:** Slawomir Swirad, Pawel Pawlus

**Affiliations:** Faculty of Mechanical Engineering and Aeronautics, Rzeszow University of Technology, Powstancow Warszawy 8 Street, 35-959 Rzeszow, Poland; ppawlus@prz.edu.pl

**Keywords:** fretting, titanium alloy, friction, wear

## Abstract

The influence of ball burnishing on friction and wear at elevated temperatures under fretting conditions has not yet been reported. Fretting experiments were conducted using the Optimol SRV5 tester (Optimol Instruments, Munich, Germany) under dry gross fretting conditions. A ball of WC ceramic was pressed against a disc from the titanium alloy Ti6Al4V. Experiments were carried out at elevated temperatures of 100, 200, and 300 °C. The displacement frequency was 50 Hz, the stroke was 0.1 mm, and the test duration was 15 min. The normal loads used were 40, 60, and 80 N. Ball burnishing led to a substantial reduction in the roughness height and an increase in the microhardness of samples from the titanium alloy. Burnishing, in most cases, caused an improvement in the friction resistance of sliding assemblies. Ball burnishing also led to wear reduction compared to the turned disc sample. The best tribological performance of the sliding pair was achieved for the disc sample burnished with the highest pressure of 40 MPa. An increase in temperature from 100 to 200 °C caused small changes in disc wear volumes and coefficients of friction. A further increase in temperature to 300 °C led to an increase in disc wear rates and friction coefficients.

## 1. Introduction

There is a need to minimise environmental pollution. Therefore, ball burnishing is an interesting alternative to abrasive machining processes, such as grinding. In ball burnishing, the ball is pressed to machined material in sliding. This process caused a decrease in surface height and an increase in hardness, leading to an improvement in the functional properties of the machine, such as resistance to wear, corrosion, and fatigue. Therefore, ball burnishing is applied in the industry, especially in aerospace and automotive engineering.

Ball burnishing is much cheaper than grinding. It can be used on the same lathe with turning, while grinding employs sophisticated machine tools and cannot be coupled with other machining processes on the same tool. Relatively low energy consumption and inexpensive tools are needed for burnishing, contrary to grinding. Ball burnishing is a chip-less process, whereas chips are generated in grinding. Grinding does not cause an increase in hardness in contrast to burnishing [1]. Therefore, burnishing often replaces grinding.

The following burnishing parameters are used: normal load, speed, and feed [2,3]. Ball burnishing is typically applied to surfaces after turning [4], milling, or grinding. It can be applied to various materials, such as steel [5,6], aluminium, or magnesium alloys [7,8]. Attabi et al. [9] and Kanovic et al. [10] achieved a substantial reduction in the height of the steel surfaces due to ball burnishing. Hardness increases due to ball burnishing were found in References [11,12]. Titanium alloys, due to their excellent functional properties, are frequently used in the biomedical, aerospace, and automotive industries [13]. Revenkar et al. [14] significantly reduced the roughness height of the titanium alloy due to ball burnishing; an increase in hardness was also achieved. Ti6Al4V is the titanium alloy most frequently applied due to its properties, such as corrosion resistance and proper mechanical behaviour at elevated temperatures [15]. Budinski [16] and Li et al. [17] experimentally studied the wear performance of Ti6Al4V samples. Ball burnishing caused an improvement in the tribological properties of sliding elements. Attabi [9] obtained a wear reduction in a steel surface due to ball burnishing. Swirad and Pawlus [18,19] achieved decreases in wear and friction after applying ball burnishing to sliding steel surfaces under dry friction conditions. Revenkar et al. [20] obtained reductions in the wear and friction coefficient of the titanium alloy in the unidirectional dry test compared to the turned surface.

Typically, tribologic tests are performed at room temperature. However, some researchers conducted the test at elevated temperatures.

The friction and wear performances of self-mated couples from ceramic using a pin-on-disc tester were studied at temperatures of 25–1000 °C. Couples’ wear levels increased with temperature [21].

Taktak [22] performed tribological tests on a borided steel disc against an Si3N4 ball at elevated temperatures (between room temperature and 600 °C). The wear rates of the steel samples increased with temperature and the steady-state friction coefficient decreased as the temperature increased.

Yang et al. [23] conducted sliding tests on the ring-on-plate configuration between WC-Co coatings and sintered alumina at temperatures of 200, 300 and 400 °C. The wear rate of the coatings decreased with increasing temperature.

Bremond et al. [24] studied the effect of temperature (between room and 400 °C) on the tribological behaviour of a DLC-coated 100C6 couple using a pin-on-disc apparatus. Coating degradation increased with temperature.

Guo et al. [25] studied the effect of the temperature (between 30 and 500 °C) of the M50 steel self-mating pairs in dry friction conditions in a ball-on-disc configuration. The friction coefficient decreased, and wear first decreased and then increased with temperature growth.

Al–Fe–V–Si alloys can be an alternative to titanium alloys to work at elevated temperatures. Koraman et al. [26] carried out the sliding tribological tests of these alloys against a ball from Al_2_O_3_ at temperatures 20–350 °C. Wear rates increased with temperature.

Tribological tests of titanium alloys were also carried out at elevated temperatures. Chi et al. [27] studied the tribological performance of a high-entropy refractory TiZrNbMo_0.6_ alloy at elevated temperatures. The results indicate that this alloy achieved optimal wear resistance at a temperature of 500 °C. A higher wear rate was found at a temperature of 800 °C than at 500 °C.

Yang et al. [28] studied the tribological behaviours of a γ-TiAl under fretting conditions at elevated temperatures. Severe adhesive wear, delamination, and abrasive wear in the early stage of fretting led to a high wear rate of the γ-TiAl alloy. As fretting progresses, the formation of nanograin-containing tribolayers and excellent mechanical properties improve the fretting wear resistance.

Yang et al. [29] investigated the role of temperature in the fretting wear of the γ-TiAl alloy. The fretting tests showed that, compared to the test at room temperature and 350 °C, a significant reduction in wear rate and a decrease in the fluctuation of the friction coefficient occurred at temperatures of 550 and 750 °C.

Although the Ti6Al4V alloy had adequate functional behaviour at elevated temperatures, researchers carried out tribological tests at higher temperatures.

Discs from Ti6Al4V and Ti6Al4V-TiC metal matrix composite coatings contacted balls from WC-Co at temperatures of 25–575 °C in the sliding motion. The wear rate and the friction coefficient decreased with increasing temperature [30].

Cui et al. [31] and Mao et al. [32] carried out dry sliding tests on Ti6Al4V pins and steel discs; the gravimetric method was used to measure the pin wear of pins at temperatures of 20 to 500 °C. Wear losses decreased as the temperature increased from 200 °C.

Zhang et al. [33] conducted sliding tests under dry friction conditions between pins of TC11 titanium alloy and steel discs at room temperature and at a temperature of 600 °C. An increase in temperature prevented wear.

Kumar et al. [34] carried out dry sliding tests on a Ti6Al4V pin and steel disc in a vacuum at temperatures of 25 to 400 °C. Wear of the titanium alloy decreased as the temperature increased.

Liang et al. [35] conducted sliding tests under dry friction conditions between the pins of WC-6Co and the Ti6Al4V disc at temperatures between 20 and 920 °C. They obtained an increase in disc wear with temperature. These results were contrary to those obtained by other researchers.

Alvi et al. [36] performed an experiment under dry friction conditions at temperatures up to 500 °C when discs were made of Ti6Al4V and balls were made of steel and alumina. However, due to the large standard deviation of the results of the wear measurement, the effect of temperature on the wear of disc from titanium alloy was difficult to assess. The coefficient of friction decreased when temperature increased.

Li et al. [37] conducted tests using a linear reciprocating tribometer with a Ti6Al4V plate and WC-Co ball at temperatures between 20 and 600 °C. Temperature has not significantly affected the wear rate of the titanium alloy.

The tribological aspects of the Ti6Al4V alloy were reviewed in [38]. Some components of turbine engines are made from this alloy, where the working temperature reaches 600 °C.

Fretting depends on the oscillating motion of two sliding surfaces at a very small amplitude. This amplitude should be smaller than the elastic contact radius [39]. Fretting can be divided into partial slip and gross slip depending on the amplitude of displacement and normal load. For a smaller amplitude of oscillation, a partial slip occurred. This kind of fretting can lead to fretting fatigue. With gross slip wear, loss occurs. The wear can have an adhesive or abrasive character [40,41].

It is evident from the literature review that the effects of the increase in temperature on the wear of samples from the Ti6Al4V alloy are conflicting. The effect of ball burnishing on friction and wear at elevated temperatures under fretting conditions has not been analysed yet. This work tries to fill this knowledge gap.

## 2. Materials and Methods

**Figure 1:**
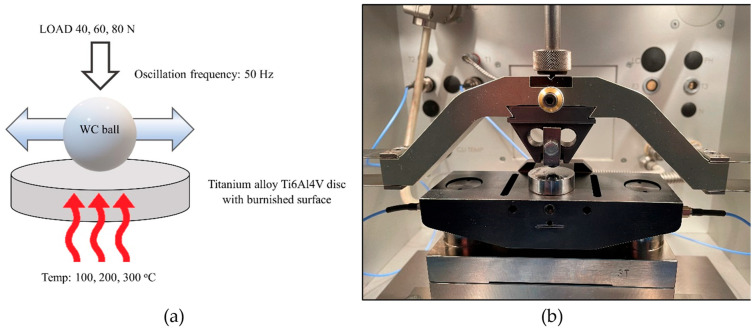
The scheme of tests (**a**) and picture of the Optimol SRV5 fretting setup (**b**).

Fretting experiments were carried out using the Optimol SRV5 tester (Optimol Instruments, Munich, Germany) in a dry reciprocating motion. The ball of 10 mm diameter made from WC ceramic was pressed against the Ti6Al4V titanium alloy disc of a 25 mm diameter and 7.9 mm height. The roughness height of the ball, determined by the Ra parameter, was 0.1 µm. Experiments were conducted at elevated temperatures of 100, 200, and 300 °C. These temperatures were selected to eliminate the possibilities of heat treatment processes. The displacement frequency of a 50 Hz stroke of 0.1 mm and test duration of 15 min were constant test parameters. Normal loads were 40, 60, and 80 N. The maximum Hertzian contact pressures for normal loads of 40, 60, and 80 N were 1470, 1685, and 1855 MPa, respectively. Each experiment was repeated three times. Humidity was set between 40 and 50%. Figure 1 shows the test scheme and picture of the Optimol SRV5 fretting setup, while Table 1 presents the test parameters.

For the turning process, the Sandvik insert cutter type CB7015 (Sandvik, Stockholm, Sweden) was used. The turning parameters were as follows: v = 125 m/min (cutting speed); f = 0.05 mm/rev (feed); and d = 0.5 mm (depth of cut). The disc samples after turning were subjected to ball burnishing with Haas CNC Vertical Mill Centre VF-1 (Haas Automation Inc., Oxnard, CA, USA). The burnishing pressures were 20, 30, and 40 MPa.

The spiral burnishing strategy provides a constant stepover relative to the burnishing surface. This technique can be used for flat surfaces and for parts with a complex curvilinear surface. The spiral burnishing strategy was used at a speed of 400 mm/min, and the burnishing width was 0.05 mm.

The ball had a diameter of 4 mm. Before tribologic tests, the surface topographies of disc samples, after turning, and ball burnishing were measured using a Talysurf CCI Lite white light interferometer (Taylor Hobson Ltd., Leicester, UK) of 0.01 nm vertical resolution. The assessment area of the surface topography measurement of 3.29 mm × 3.29 mm contained 1024 × 1024 data points. Before computing parameters, the measured topographies were levelled, and non-measured points were filled in. Digital filtration was not used. The wear of the co-acting parts was measured using a profilometric method, also using Talysurf CCI Lite. The volume of the wear scar on the disc surface was determined based on the measurement of areal (3D) surface topography. The surface textures were levelled, excluding wear scars. Next, the non-measured points were filled in with spike elimination when necessary. Then, the volumes of the scars were calculated [42]. During the tests, the coefficient of friction was recorded as a function of time. The following parameters characterising disc surface textures were selected: rms, height Sq, skewness Ssk, kurtosis Sku, peak height Sp, valley depth Sv, maximum height Sz, average height Sa, core depth Sk, reduced peak height Sp, reduced valley depth Svk, correlation length Sal, and texture aspect ratio Str. Sq, Sp, Sv, Sz, Sa, Sk, Spk, and Svk are amplitude parameters; Ssk and Sku characterise the surface ordinate distribution [43]; and Sal and Str are spatial parameters.

Microhardness measurements of the disc samples after burnishing and turning, as well as after tribologic tests, were carried out using Reicherter Brivisor KL2 Vickers indenters (Buehler Ltd., Lake Bluff, IL, USA). The maintenance time at the maximum load was 25 s.

## 3. Results and Discussion

Table 2 lists the values of the parameters selected according to the ISO 25178-2:2021 [44] standard for turned and burnished disc samples. The surface, after turning, had an anisotropic (Str = 0.14) periodic character (Sku smaller than three; view of the surface). Due to burnishing, the roughness amplitude decreased, and this decrease was the highest after applying the medium burnishing pressure. When the other pressures were applied, the surface height was similar. Burnishing caused an increase in the kurtosis Sku. The correlation length Sal increased as a result of burnishing; the highest growth occurred for the smallest burnishing pressure. The texture aspect ratio Str increased as a result of burnishing; the character of the surface changed from anisotropic to mixed. Skewness decreased after the use of the medium burnishing pressure; in the other cases of burnishing, it increased.

**Figure 2:**
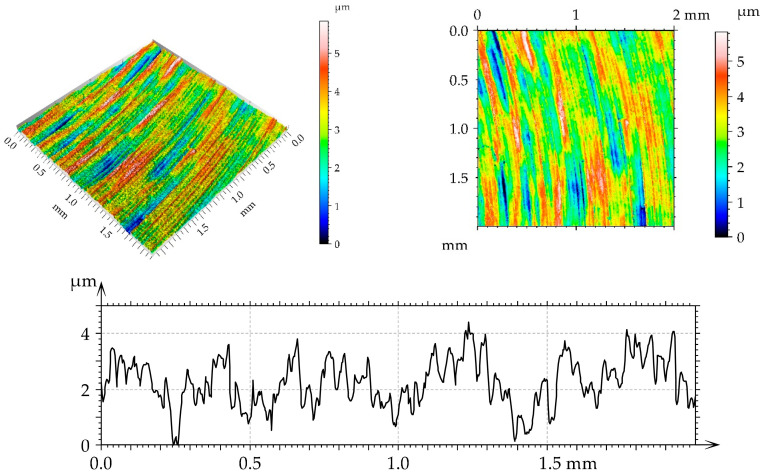
Isometric view, contour plot, and extracted profile of the disc surface after turning.

**Figure 3:**
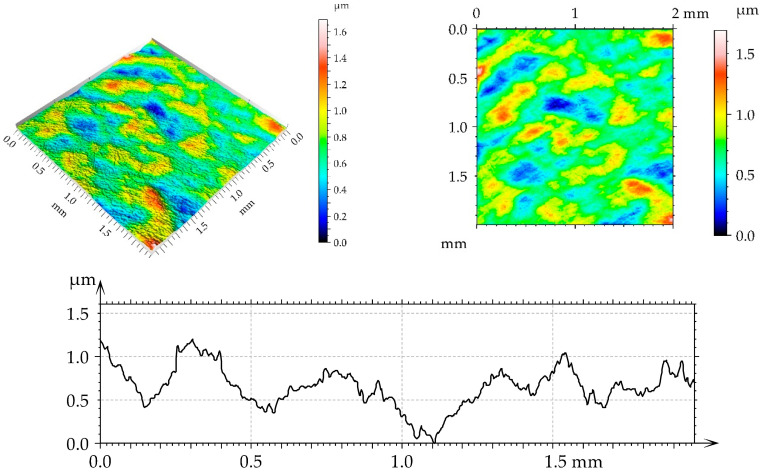
Isometric view, contour plot, and extracted profile of the disc surface after spiral burnishing, with a pressure of 20 MPa.

**Figure 4:**
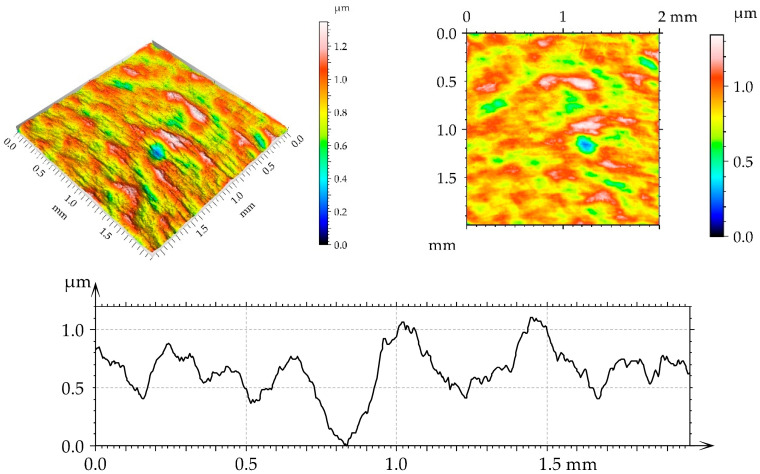
Isometric view, contour plot, and extracted profile of the disc surface after spiral burnishing, with a pressure of 30 MPa.

**Figure 5:**
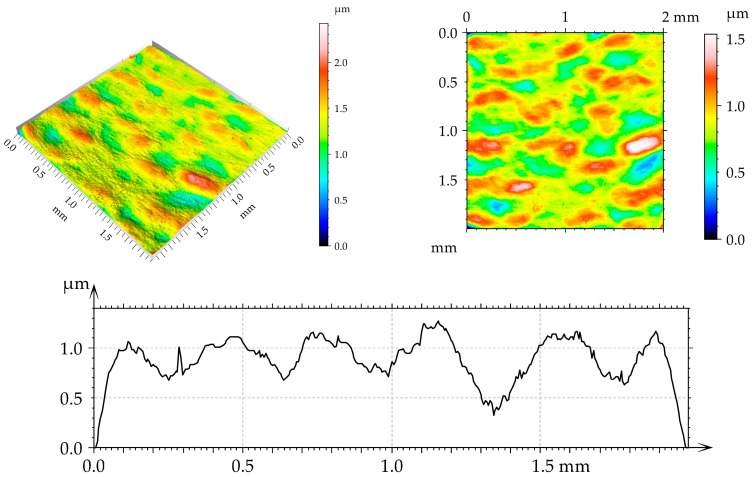
Isometric view, contour plot, and extracted profile of the disc surface after spiral burnishing, with a pressure of 40 MPa.

Figure 2, Figure 3, Figure 4 and Figure 5 present isometric views, contour plots, and representative profiles of the disc surfaces. The surface texture disc surface after turning (Figure 2) had a periodic, anisotropic character. Circular grooves can be seen in isotropic views and contour plots of this surface. These grooves are not observed on the burnished surfaces of the mixed structure (Figure 3, Figure 4 and Figure 5). The burnished surface had a smaller amplitude than the turned texture, and a burnishing pressure of 30 MPa led to the lowest height (Figure 4).

**Figure 6:**
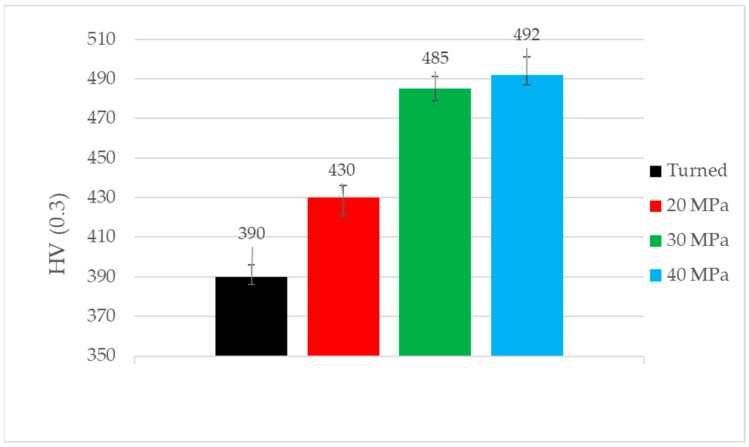
Microhardness values of discs before tribologic tests.

Figure 6 presents the results of the microhardness measurement before tribologic tests. Burnishing caused an increase in the microhardness of the discs. The highest changes occurred for burnishing pressures of 30 and 40 MPa. The highest increase in microhardness for a burnishing pressure of 40 MPa was 26%, while the smallest increase for a pressure of 20 MPa was 10%.

**Figure 7:**
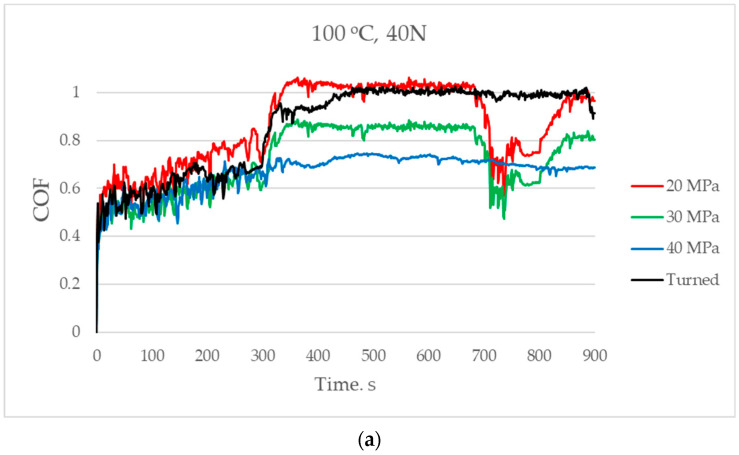

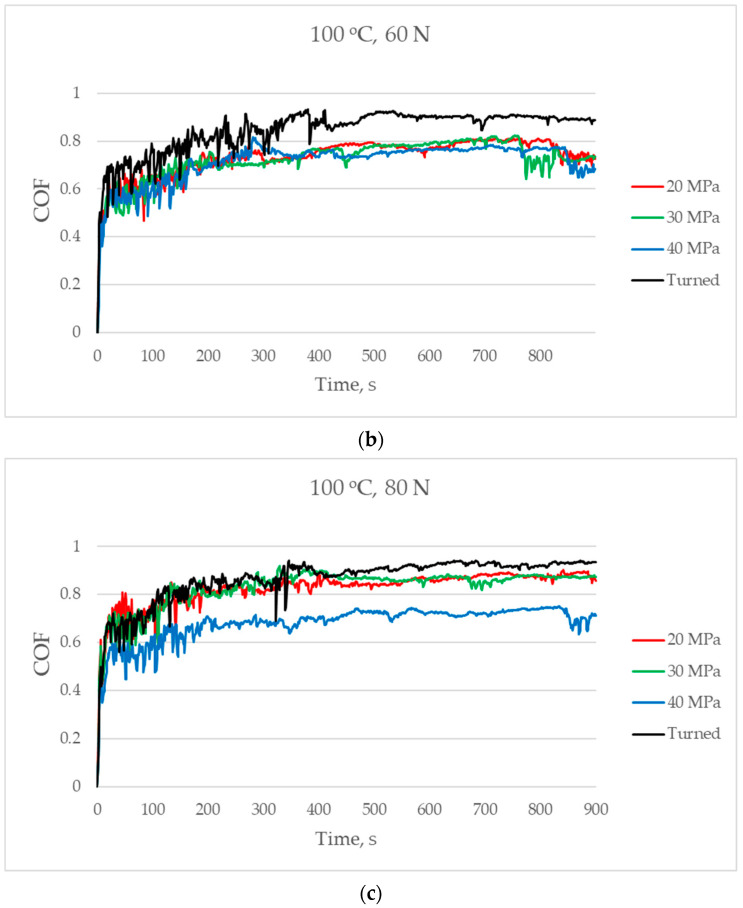
The coefficient of friction versus time for temperature at 100 °C and for normal loads of 40 (**a**), 60 (**b**), and 80 N (**c**).

Figure 7 presents the changes in the coefficient of friction versus time for a temperature of 100 °C. Independently of the load, friction forces increased and stabilised after 300–400 s. For the smallest normal load of 40 N, the smallest coefficient of friction occurred for sliding pairs with the disc sample burnished and the highest pressure followed by the medium pressure. A sudden step at 300 s was probably related to the settlement of the wear debris inside wear scars. Rapid friction coefficient reductions between 700 and 850 s for two sliding pairs with burnished samples (pressures 20 and 30 MPa) may be caused by the settlement of wear debris in unhardened layers of the disc surfaces.

For the normal load of 60 N, the highest coefficient of friction corresponded to the assembly with the turned sample. The turned disc also led to the highest coefficient of friction for the highest normal load of 80 N; in this case, the smallest coefficient of friction corresponded to the highest burnishing pressure. Generally, the lowest frictional resistance was achieved for the sliding pair containing the burnished disc sample with the highest pressure; however, the highest friction was achieved for the assembly with the turned sample. The coefficients of friction in the final part of the test were between 0.7 and 1 for the smallest normal load applied; in the other cases, they were between 0.7 and 0.9.

**Figure 8:**
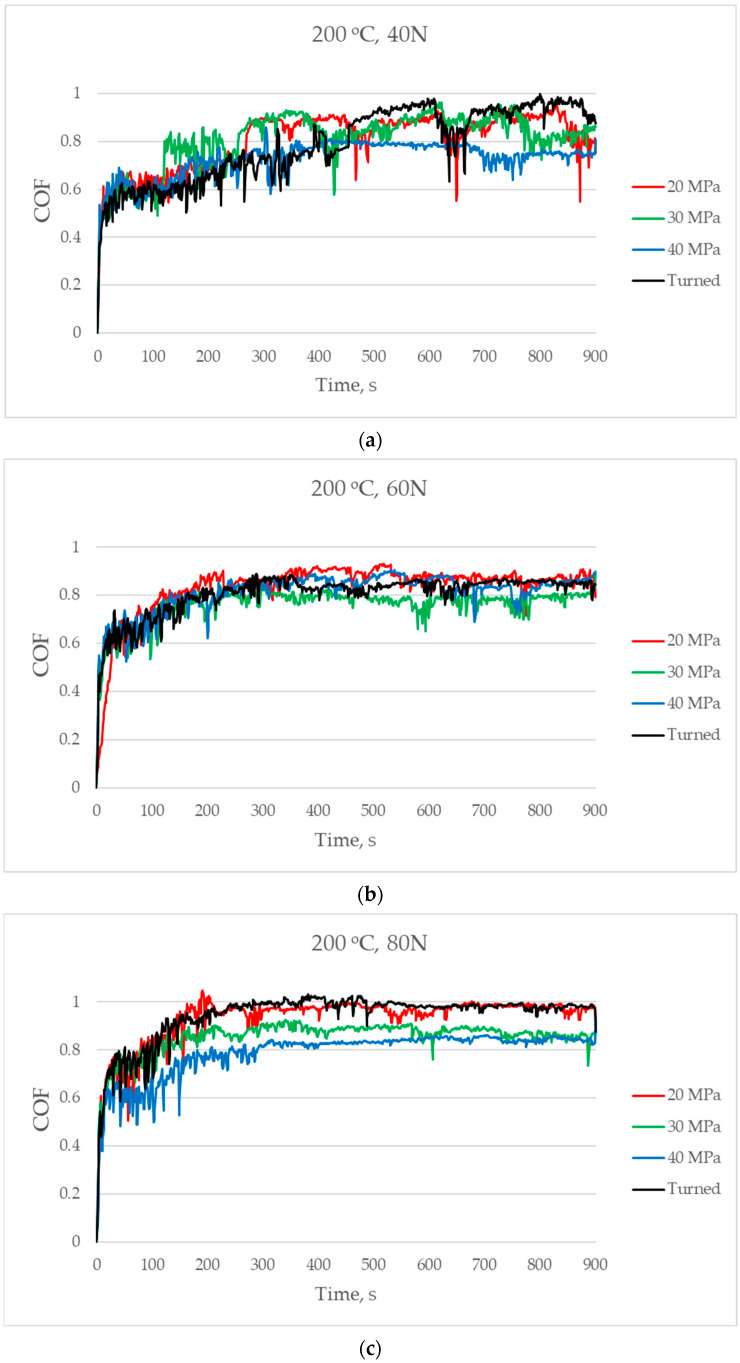
The coefficient of friction versus time for a temperature of 200 °C and for normal loads of 40 (**a**), 60 (**b**), and 80 N (**c**).

Figure 8 presents the changes in the coefficient of friction versus time for a temperature of 200 °C. The friction force after initial growth was stabilised later when the load was diminished: for a load of 40 N after 500 s, for a load of 60 N after 400 s, and for a load of 80 N after 300 s, approximately. For the smallest applied normal load, the burnished sample with the highest pressure resulted in the smallest friction coefficient. The final friction coefficient was between 0.8 and 1. When the medium normal load was applied, the friction coefficients were similar for all sliding pairs—the final scatter was between 0.8 and 0.9. In the final part of the test, the smallest coefficient of friction was achieved for the sliding assembly with the disc sample burnished with a pressure of 30 MPa. For the highest normal load, the smallest coefficient of friction was achieved for the sliding pair with the disc sample burnished with the highest pressure, followed by the sample burnished with the medium pressure. In this case, similar to the smallest normal load, the coefficient of friction was between 0.8 and 1.

**Figure 9:**
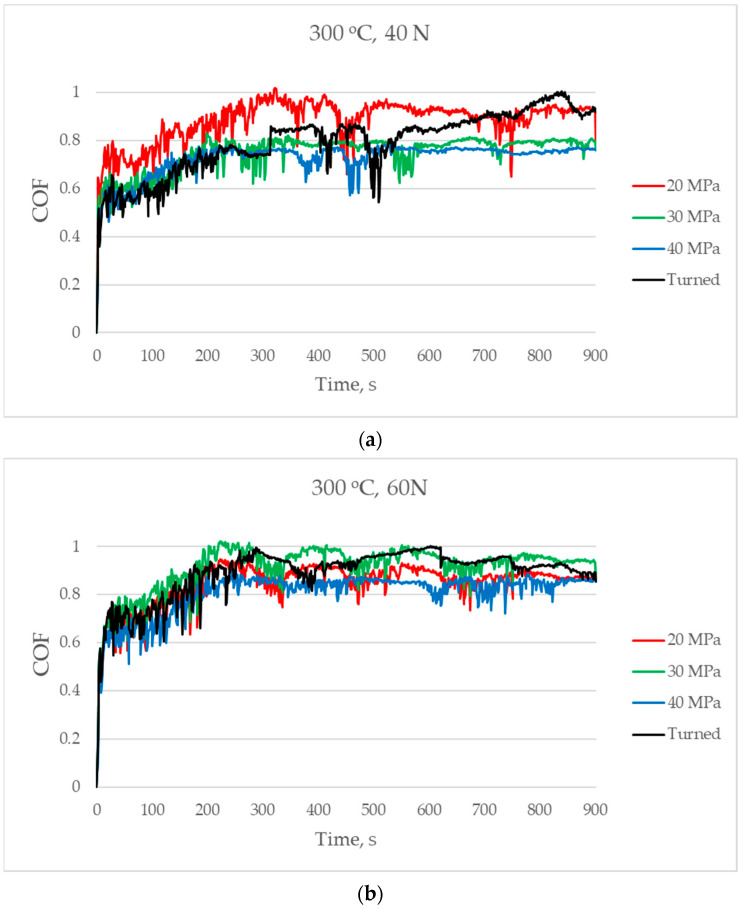

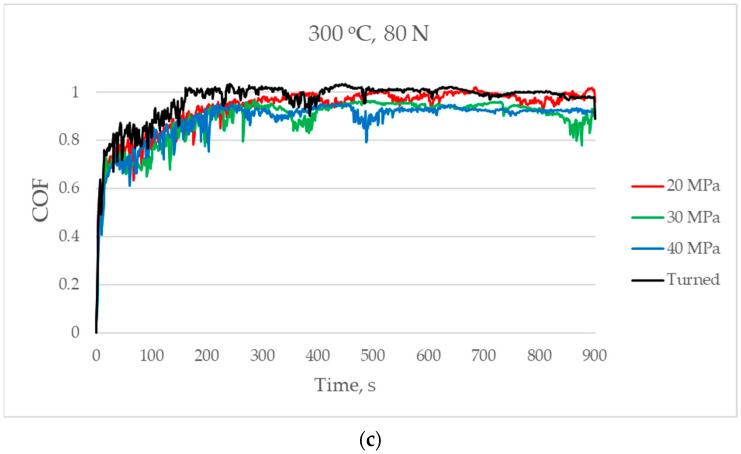
The coefficient of friction versus time for a temperature of 300 °C and for normal loads of 40 (**a**), 60 (**b**), and 80 N (**c**).

Figure 9 presents the changes in the coefficient of friction versus time for a temperature of 300 °C. The friction force was stabilised similarly to work at a temperature of 200 °C. In the final parts of the tests, the coefficient of friction reached values between 0.8 and 1, independently of the normal load used. For the smallest normal load, the smallest coefficients of friction were achieved for sliding pairs that had a burnished sample with pressures of 40 and 30 MPa. When medium and highest normal loads were used to create disc samples, the lowest coefficient of friction was achieved for the burnished disc with a pressure of 40 MPa, but the highest was achieved for the turned disc.

**Figure 10:**
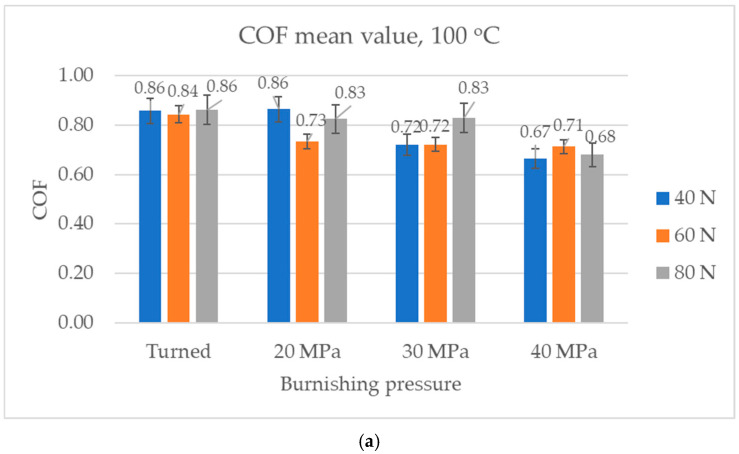

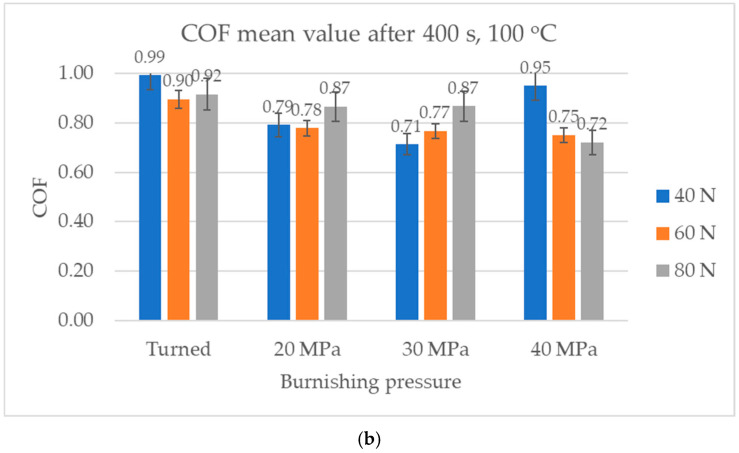
Average values and error bars of the coefficient of friction obtained at a temperature of 100 °C: average (**a**) and in the final part of test (**b**).

Figure 10 presents the average values and error bars (standard deviations) of the coefficient of friction obtained at the smallest temperature of 100 °C. Similar tendencies were obtained throughout the tests (Figure 10a) and in the final parts of the tests (Figure 10b), except for the burnished sample with the highest pressure of 40 MPa and the smallest normal load of 40 N (mean values in the entire tests were the smallest, while in the final part of the test, they were high). For the smallest normal load, mean values were lower for burnished samples obtained with pressures of 30 and 40 MPa compared to other sliding pairs. Under the same conditions, burnished samples with pressures of 20 and 30 MPa caused a smaller amount of friction than other samples throughout the test. When the normal load of 60 N was applied, the highest coefficients of friction were obtained for the assembly with the turned disc sample. However, for the highest normal load, sliding the disc sample burnished with the highest pressure led to the smallest coefficient of friction.

**Figure 11:**
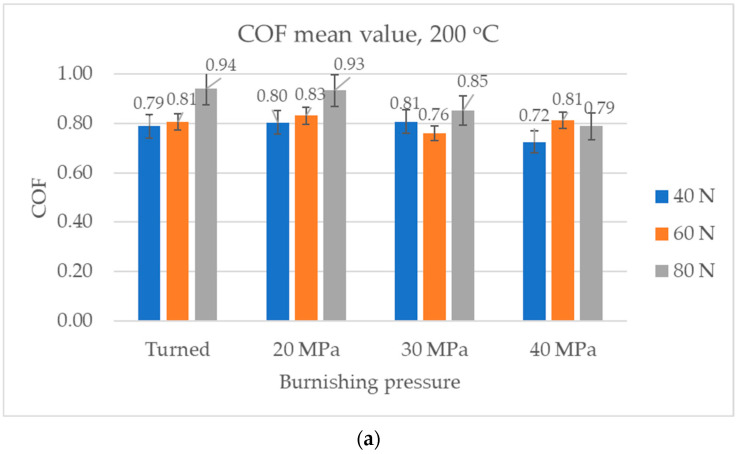

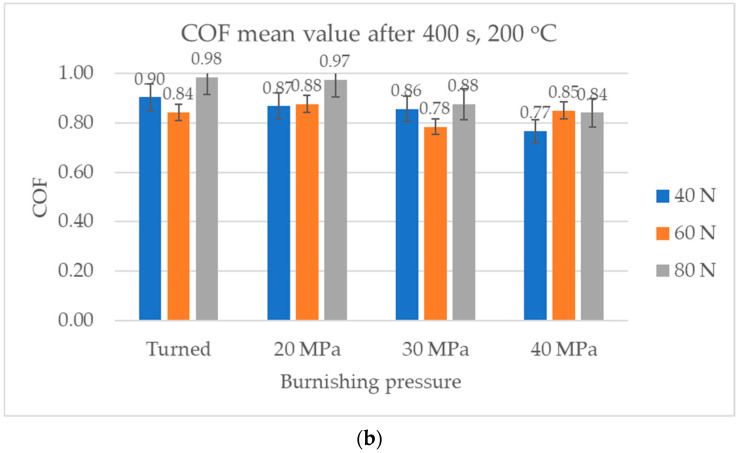
Average values and error bars of the coefficient of friction obtained at a temperature of 200 °C: average (**a**) and in the final part of test (**b**).

Figure 11 presents the average values and error bars of the coefficient of friction at the smallest temperature of 200 °C. Under these conditions, similar results were obtained throughout the test and in its final part. For the smallest normal load, the sliding pair with the burnished sample with the highest pressure obtained a smaller coefficient of friction compared to other assemblies. When the normal load was 60 N, the coefficient of friction in the final test part corresponding to the burnished sample with a pressure of 30 MPa was smaller compared to those of burnished samples with other pressures. For the highest normal load, the sample burnished with the highest pressure led to a smaller coefficient of friction than the turned disc and burnished disc with the smallest pressure of 20 MPa.

**Figure 12:**
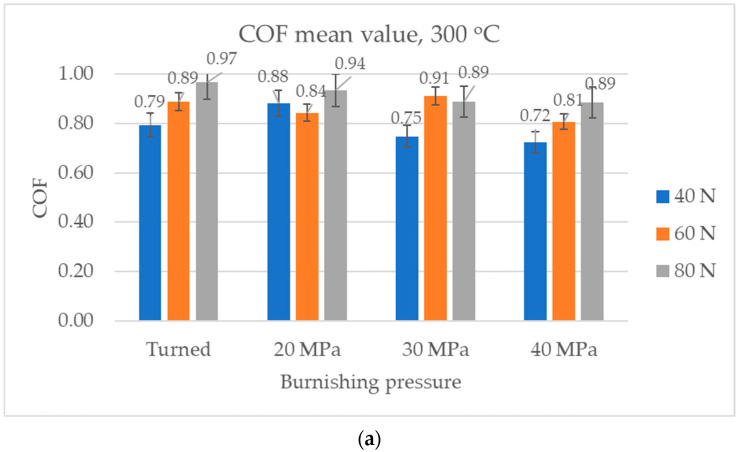

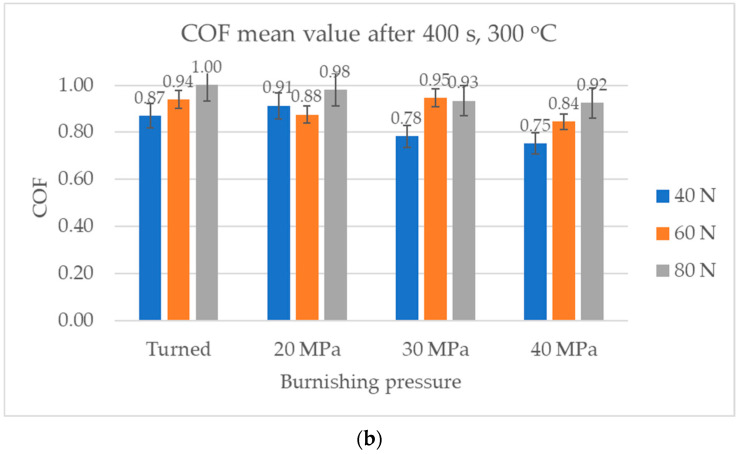
Average values and error bars of the coefficient of friction obtained at a temperature of 300 °C: average (**a**) and in the final part of test (**b**).

Figure 12 presents the average values and error bars of the coefficient of friction at the smallest temperature of 300 °C. A similar tendency of friction occurred throughout the test and in the final parts of the test. For the smallest normal load applied, the coefficients of friction obtained for samples burnished with pressures of 30 and 40 MPa were considerably smaller compared to the other samples. When the normal load was 60 N, the smallest coefficients of friction were achieved for sliding pairs with burnished samples and pressures of 20 and 40 MPa. For the highest normal load applied, no significant differences were found between the frictional resistances obtained and the tested assemblies were not found.

**Figure 13:**
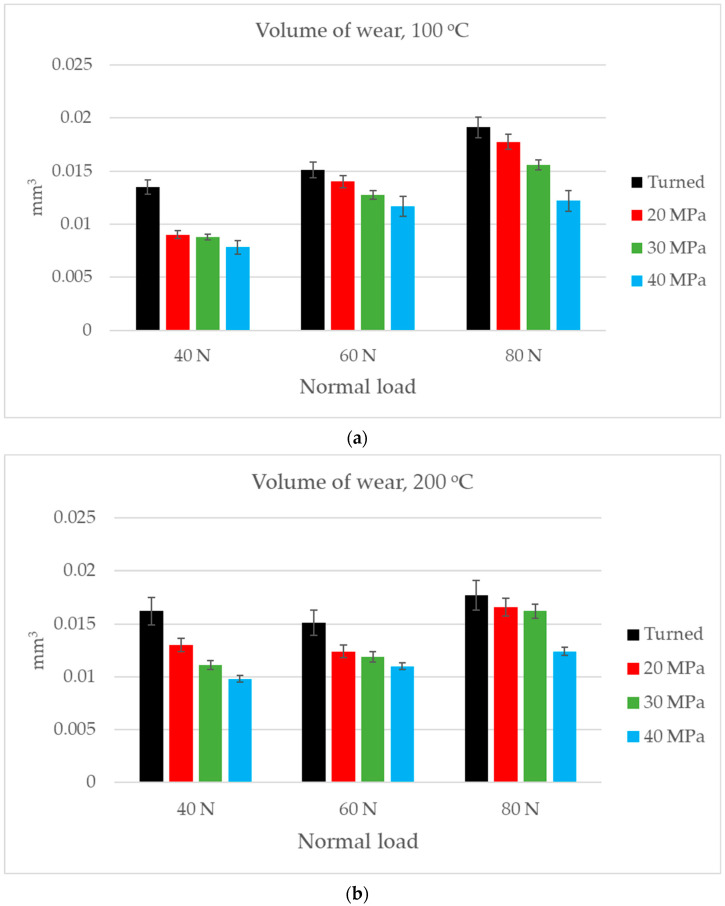

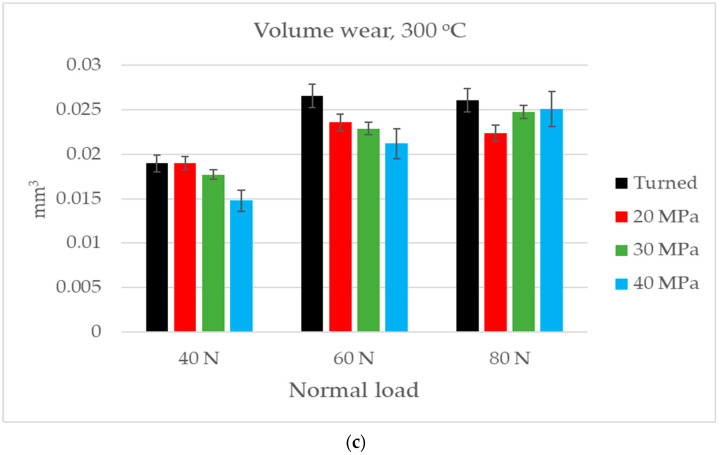
Wear volumes of tested discs at temperatures of 100 (**a**), 200 (**b**), and 300 °C (**c**).

The wear values of the balls were much lower than those of the discs because the hardness of the ball was considerably higher than the hardness of the disc. Figure 13 presents the mean value and error bars of the volumes of disc wear.

When the experiment was carried out at a temperature of 100 °C (Figure 13a) for the normal load of 40 N, the wear rate of the turned disc was considerably higher than that of other samples; the smallest volumetric wear was achieved on the disc sample burnished with a pressure of 40 MPa. For the medium normal load applied, the wear volumes of the turned sample and the disc burnished with a pressure of 20 MPa were higher than those of the other discs. When the normal load was the highest, the smallest wear was achieved for the disc burnished with a pressure of 40 MPa, followed by the sample burnished with a pressure of 30 MPa.

For a temperature of 200 °C and normal loads of 40 and 60 N, the wear volumes of the turned samples were the highest. When the normal load was 40 N, the smallest volumetric wear was achieved for the burnished sample with a pressure of 40 MPa, followed by that of the burnished sample with a pressure of 30 MPa. For a normal load of 60 N, the volumetric wear of the disc burnished with a pressure of 40 MPa was less than the wear levels of other burnished discs. When the highest normal load was applied, the smallest wear was obtained for the burnished disc with a pressure of 40 MPa, followed by the burnished disc with a pressure of 30 MPa (Figure 13b).

When the experiment was conducted at the highest temperature of 300 °C, the volumetric wear of the turned disc was the highest for the medium normal load. For the smallest and medium normal loads, the lowest wear volumes were achieved for the disc burnished with a pressure of 40 MPa, but the highest normal load was achieved for the disc burnished with a pressure of 30 MPa (Figure 13c).

**Figure 14:**
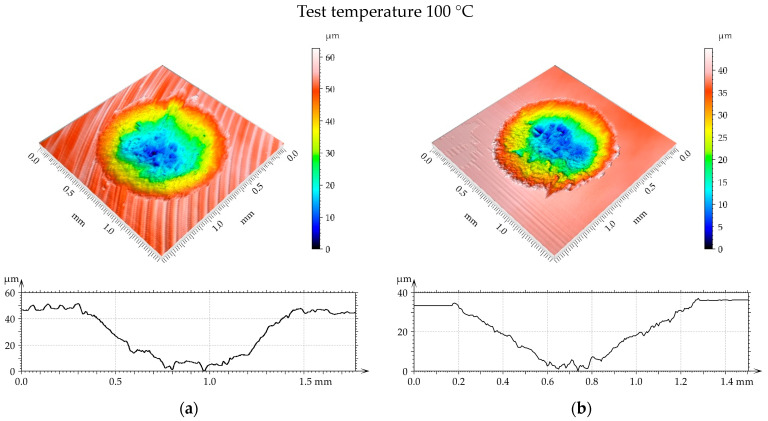
Example of disc wear at different test parameters: (**a**) turned with load of 40 N and wear volume of 0.0135 mm^3^ and (**b**) burnished with pressure of 30 MPa, load 40 N, and wear volume 0.008 mm^3^.

**Figure 15:**
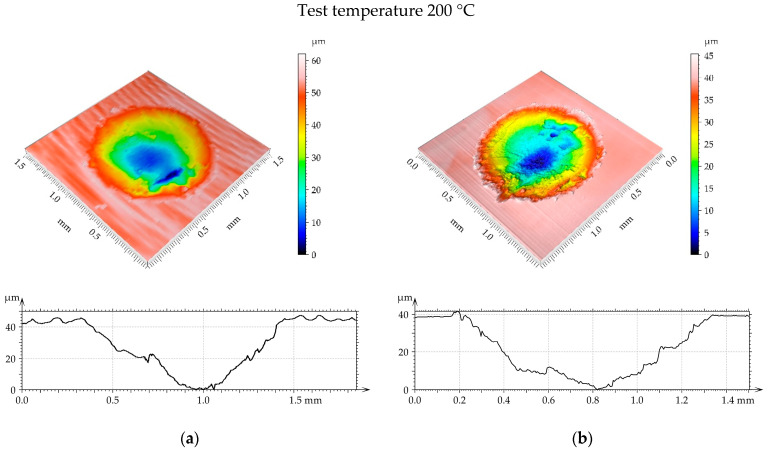
Example of disc wear at different test parameters: (**a**) turned with load of 60 N and wear volume of 0.015 mm^3^ and (**b**) burnished at pressure of 40 MPa, load 60 N, and wear volume 0.011 mm^3^.

**Figure 16:**
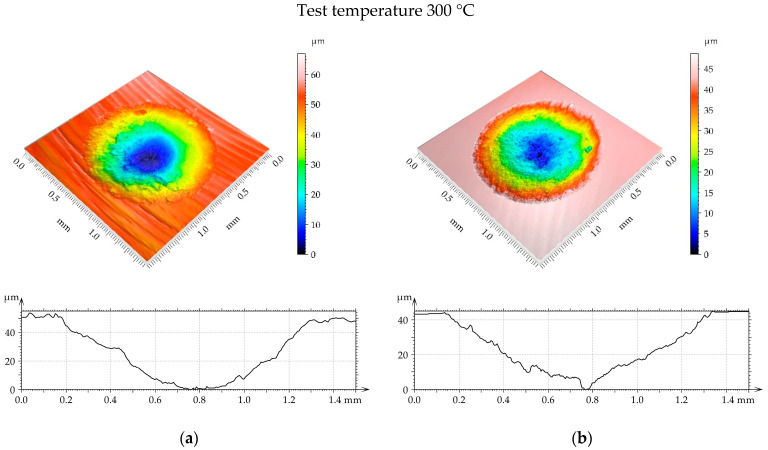
Example of disc wear at different test parameters: (**a**) turned with load of 80 N and wear volume of 0.027 mm^3^ and (**b**) burnished with pressure of 40 MPa, load 80 N, and wear volume 0.024 mm^3^.

Figure 14, Figure 15 and Figure 16 present isometric views of examples of worn discs. A gross slip occurred and the wear had an abrasive character because the cross sections of the wear scar had shapes of the “U” letter. For a temperature of 100 °C (Figure 14), the diameter and depth of the wear scar were higher for the turned disc compared to the burnished sample. When the temperature was 200 °C (Figure 15), the linear and volumetric wear levels of the turned disc were higher than those of the burnished sample. A similar situation occurred for the highest temperature of 300 °C (Figure 16). In this case, the linear wear levels were higher compared to those obtained for lower temperatures (Figure 14 and Figure 15).

**Figure 17:**
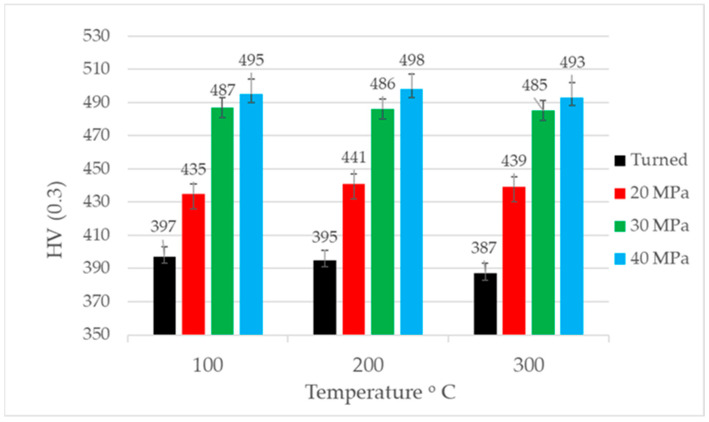
Microhardness values of discs after tribologic tests at normal load of 80 N.

Figure 17 presents the microhardness values of the discs after tribologic tests at a normal load of 80 N. These values were similar to those obtained after machining (Figure 6). In most cases, the microhardness marginally increased; the highest growth was 11 MPa (2.5%). Very similar values were obtained after tests at smaller normal loads.

The amount of heat released from friction, no higher than 720 Joules, did not substantially affect the results of the tribological tests, taking into account the construction of the tester and the dimensions of the disc sample.

As a result of ball burnishing, the roughness height considerably decreased, and the character of the surface changed from anisotropic to mixed (the Str parameter increased). The lowest roughness height was achieved for a burnishing pressure of 30 MPa. Ball burnishing also caused an increase in the microhardness values of the disc samples. The smallest burnishing pressure led to the lowest microhardness increase. The highest increase in microhardness was obtained for the highest burnishing pressure. Similar changes were found in other works [18,19,45].

The courses of the coefficient of friction versus time were similar for operating at various temperatures. The friction initially increased significantly and obtained a stable value. For temperatures of 200 and 300 °C, the coefficient of friction stabilised faster for a higher normal load. Perhaps surfaces of co-acting parts matched faster for larger normal loads.

The obtained coefficients of friction seem to be high. However, similar values were obtained in other research under dry gross fretting conditions. Lenart et al. [46] obtained coefficients of friction up to 0.75 in contact with the steel disc with the ceramic ball under gross fretting conditions at a temperature of 30 °C. Kubiak et al. [47] acquired a coefficient of friction near 0.95 for dry contact between the steel ball and the plate of the titanium alloy. Wiener et al. [48] obtained the friction coefficient near one at temperatures of 23 and 100 °C. Llavori et al. [49] found that the maximum friction coefficient was sometimes higher than one. Podgursky et al. [50] obtained a friction coefficient close to one in the dry fretting tests.

Generally, ball burnishing caused an improvement in the friction resistance of sliding assemblies. The smallest coefficient of friction was achieved for the sample obtained with the highest burnishing pressure of 40 MPa, followed by a pressure of 30 MPa. A significant decrease in the coefficient of friction as a result of ball burnishing with the smallest pressure was found compared to the assembly with a turned disc only for the smallest temperature. The decrease in the coefficient of friction due to ball burnishing was probably caused by a reduction in the roughness height. For the highest temperature and normal load only, the effect of ball burnishing on changes in the friction force compared to the behaviour of assembly with the turned disc sample was negligible.

The friction coefficients at temperatures of 100 and 200 °C were similar. Higher temperatures led to a higher coefficient of friction. The behaviour of the coefficient of friction was similar to that of volumetric wear. A lower coefficient of friction corresponded to a smaller wear volume.

The effects of ball burnishing on disc wear were similar at temperatures of 100 and 200 °C. This effect at 300 °C was visible only for the normal load of 60 N. Burnishing caused a wear reduction compared to the turned disc sample. This reduction was higher when the burnishing pressure was also higher. This behaviour was probably caused by the combination of the decrease in roughness height and increase in microhardness due to ball burnishing. However, the last impact seems to be more important. Similar behaviour was found in other investigations [18,19]. When the temperature was the highest, and the normal load was the smallest, the differences between the wear volumes of the turned and burnished samples with the smallest pressure disc samples were not substantial. At the highest temperature and normal load, the wear of a burnished sample with a pressure of 20 MPa was the smallest. In these conditions, the differences among the friction coefficients were negligible.

An increase in temperature from 100 to 200 °C caused small changes in the volumetric wear levels of the disc samples. Li et al. [37] also found that temperature did not significantly affect the wear rate of the sample made from the titanium alloy. In this work, a further increase in temperature led to an increase in disc wear volumes. Similar results were obtained by Liang et al. [35]. However, the authors of References [30,31,32,33,34] obtained opposing results.

It is evident from the analysis of Figure 14, Figure 15 and Figure 16 that the disc wear had an abrasive character. A gross slip occurred.

The increase in temperature had a negligible effect on disc microhardness. Microhardness can decrease with temperature growth [33]. Probably, in this research, the temperature rise was too small to affect the microhardness.

In this research, it was found that ball burnishing is an interesting alternative to turning. In most cases, the burnishing of the titanium alloy caused an improvement in the friction resistance of sliding assemblies at elevated temperatures.

## 4. Conclusions

1. The ball burnishing led to a substantial reduction in the roughness height of the samples from Ti6Al4V. The use of a burnishing pressure of 30 MPa led to the smallest texture amplitude. Ball burnishing also caused an increase in microhardness. The highest microhardness growth was obtained for the highest burnishing pressure of 40 MPa.
2. The ball burnishing of the samples of titanium alloy, in most cases, caused an improvement in the frictional resistance of sliding assemblies. Typically, the highest friction reduction was reached for the burnished disc with a pressure of 40 MPa, followed by a pressure of 30 MPa. A significant decrease in the coefficient of friction due to ball burnishing with the smallest pressure of 20 MPa was obtained only for the smallest working temperature of 100 °C.
3. Ball burnishing caused wear reduction compared to the turned disc sample. This decrease was greater when the burnishing pressure was greater at temperatures of 100 and 200 °C. This tendency at 300 °C was visible only for the normal load of 60 N. At the highest temperature and a normal load of 80 N, the wear volume of the sample burnished with a pressure of 20 MPa was the smallest.
4. An increase in temperature from 100 to 200 °C caused small changes in the volumetric wear levels of the disc samples and the coefficients of friction. A further increase in the temperature to 300 °C led to an increase in the disc wear volumes and friction coefficients.
5. Temperature increases during tribologic tests had a negligible effect on the disc microhardness.

## Figures and Tables

**Table 1:** Fretting test parameters.

Parameter	Value
Frequency	50 Hz
Humidity	40–50%
Stroke	0.1 mm
Normal load	40, 60, 80 N
Time	15 min
Sliding distance	9 m
Sample temperature	100, 200, 300 °C

**Table 2:** Values of selected areal surface texture parameters of tested disc samples.

			Burnished		
Parameters		Turned	20 MPa	30 MPa	40 MPa
Sq	µm	1.04	0.200	0.136	0.186
Ssk		−0.0385	0.110	-0.103	0.183
Sku		2.89	3.14	3.75	3.81
Sp	µm	2.80	1.00	0.468	1.10
Sv	µm	3.03	1.56	0.675	1.66
Sz	µm	5.84	2.57	1.14	2.76
Sa	µm	0.832	0.159	0.105	0.145
Sk	µm	2.40	0.436	0.347	0.400
Spk	µm	0.912	0.152	0.155	0.149
Svk	µm	0.914	0.161	0.166	0.110
Sal	mm	0.0494	0.118	0.085	0.0888
Str		0.136	0.535	0.532	0.370

## Data Availability

The original contributions presented in the study are included in the article, further inquiries can be directed to the corresponding author.
